# Non-Motor Symptoms as Predictors of Quality of Life in Egyptian Patients With Parkinson’s Disease: A Cross-Sectional Study Using a Culturally Adapted 39-Item Parkinson’s Disease Questionnaire

**DOI:** 10.3389/fneur.2018.00357

**Published:** 2018-05-24

**Authors:** Ali S. Shalash, Eman Hamid, Hanan Hani Elrassas, Ahmed Safwat Bedair, Abdelrahman Ibrahim Abushouk, Mohamed Khamis, Mostafa Hashim, Nahed Salah-Eldin Ahmed, Samia Ashour, Mahmoud Elbalkimy

**Affiliations:** ^1^Department of Neurology, Faculty of Medicine, Ain Shams University, Cairo, Egypt; ^2^Faculty of Medicine, Okasha Institute of Psychiatry, Ain Shams University, Cairo, Egypt; ^3^Faculty of Medicine, Ain Shams University, Cairo, Egypt

**Keywords:** 39-item Parkinson’s disease questionnaire, Arabic, Egypt, non-motor, Parkinson’s disease, quality of life

## Abstract

**Objective:**

The prevalence of non-motor symptoms (NMSs) and their impact on health-related quality of life (HRQoL) in Parkinson’s disease (PD) has been reported inconsistently among different populations. In this study, we aimed to investigate the NMSs and HRQoL profiles and their correlation in Egyptian PD patients, using a culturally adapted Arabic version of the 39-item Parkinson’s disease questionnaire (PDQ-39).

**Methods:**

Ninety-seven PD patients were rated using the unified Parkinson’s disease rating scale (UPDRS), the non-motor symptoms scales (NMSS), Beck depression inventory (BDI), and the Arabic version of PDQ-39. We used the Spearman’s rank correlation and multiple linear regression analyses to evaluate the relationship between NMSs domains and HRQoL dimensions.

**Results:**

Fatigue/sleep (91.3%) and mood/cognitive disturbances (87%) were the most frequently and severely affected NMSS domains. Other common NMSs included urinary (75.9%), memory/attention (72.4%), gastrointestinal (67.8%), and cardiovascular problems (64.8%). The total NMSS scores were positively correlated with UPDRS I, II, and III scores. Depression was prevalent in 76.7% of PD patients. Moreover, all enrolled PD patients reported impairment in different HRQoL dimensions, especially mobility (98.9%), activities of daily living (97.8%), and emotional well-being (95.5%). The summary index of PDQ-39 was correlated to the total NMSS, UPDRS-I, UPDRS-II Off, UPDRS-III (Off and On states), and BDI scores.

**Conclusion:**

This study showed the high prevalence of NMSs and the value of NMSS and BDI scores as predictors of HRQoL in Egyptian PD patients. Therefore, characterizing the NMSs profile is essential for tailoring management strategies for PD patients.

## Introduction

Parkinson’s disease (PD) is the second most common neurodegenerative disorder worldwide, caused by degeneration of dopaminergic neurons in the substantia nigra, and affects approximately 1–3% of the elderly population (≥60 years) (1). Health-related quality of life (HRQoL) is a multidimensional, self-reported measure of the disease impact on the patients’ lives. Improving the HRQoL is the aim of care in chronic diseases, especially PD in which HRQoL is determined by motor, non-motor symptoms (NMSs), and other social factors (2).

The NMSs consist of autonomic dysfunction, sensory symptoms, neuropsychiatric disturbances, sleep problems, fatigue, and gastrointestinal (GIT) disorders. Their impact on HRQoL was reported to be greater than the motor symptoms of PD (3). However, the prevalence of NMSs and their influence on HRQoL in PD patients had been shown to vary among different countries and cultures (4–8). For example, constipation and cognitive deficit were highly prevalent in Asian (especially Chinese) patients (8), compared with Western countries. Moreover, another study showed that bodily discomfort and stigma were the most impaired HRQoL domains among Chinese PD patients, in contrast to other populations (9). These differences could be attributed to several demographic, genetic, and clinical variations in PD patients (10). Moreover, different NMS profiles were identified, resulting in the proposal of NMS subtypes of PD (11). Therefore, identifying the prevalent NMS subtype in various settings will help to personalize the management of PD patients (12).

Despite the high prevalence and genetic variability of PD in Egyptian and Arab populations (13–15), only few studies investigated the NMSs profile in Egyptian PD patients and none—to the best of our knowledge—explored their value as predictors of HRQoL (16, 17). The prevalence of PD among the Egyptian population is the highest compared with other Arabic countries, which may be due to genetic or environmental factors (14, 18). Providing a culturally adapted Arabic version of the 39-item Parkinson’s disease questionnaire (PDQ-39) would facilitate epidemiological studies and improve patients’ care.

Therefore, this study aimed to explore the NMS and HRQoL profiles and their correlations in Egyptian PD patients. Furthermore, it provides a culturally adapted Arabic version of the PDQ-39 scale.

## Materials and Methods

### Patients

Ninety-seven patients diagnosed with idiopathic PD were recruited from the movement disorders outpatient clinic at Ain Shams University Hospitals (Cairo, Egypt) between 2013 and 2017. The recruited patients were diagnosed according to the British Parkinson’s disease Society Brain Bank criteria (19). Patients with atypical or secondary Parkinsonism and those with other comorbid chronic diseases that could affect PD NMSs and/or HRQoL were excluded. Moreover, PD patients who refused or could not complete questionnaires, e.g., due to severe cognitive impairment or being on antidepressants were excluded. The Ethical Committee at the Faculty of Medicine, Ain Shams University approved the study protocol. All subjects gave written informed consent according to the Declaration of Helsinki (1975, as revised in 2008).

### Outcome Measurement

All subjects were evaluated using the unified Parkinson’s disease rating scale (UPDRS), Hoehn and Yahr scale (H&Y), and Schwab and England scale (S&E) in medication “off” and “on” states by a movement disorders expert. Different UPDRS subscales were estimated, including cognition (UPDRS-I), activities of daily living (ADL, UPDRS-II), motor (UPDRS-III), and total UPDRS scores. Moreover, depression was assessed using the Beck depression inventory (BDI) (20).

The NMSs were assessed in all patients, using the non-motor symptoms scale (NMSS) (21), which consists of 30 items, grouped in nine domains [cardiovascular (CVS), sleep/fatigue, mood/cognition, perception/hallucinations, memory/attention, GIT, urinary, sexual, and miscellaneous symptoms]. The frequency of each NMS (item) was calculated (item score ≥ 1), and the summary index for each domain was estimated (the sum of included items divided by the maximum possible score then multiplied by 100) to allow crude comparisons between the severity of different domains.

The HRQoL of PD patients was measured using a culturally adapted Arabic version of the Parkinson’s disease questionnaire (PDQ-39) (22), which is formed of 39 items (ranged from 0 = never to 4 = always) that are grouped into eight dimensions. These dimensions include mobility (1–10, 10 items), ADL (11–16, 6 items), emotional well-being (17–22, 6 items), stigma (23–26, 4 items), social support (27–29, 3 items), cognition (30–33, 4 items), communication (34–36, 3 items), and bodily discomfort (37–39, 3 items). Following answering the questionnaire, a summary index for each dimension (subscales) was calculated by dividing the sum of included items by the maximum possible score then multiplying by 100. Then, the total score [PDQ-39 summary index (PDQ-39 SI)] was calculated by summation of the eight dimensions’ scores divided by 8. Consequently, lower scores reflect better HRQoL (23). The frequency of impairment of each dimension to any degree (score > 0) was also estimated.

### PDQ-39 Cultural (Same Language) Adaptation

After a written agreement from the scale provider (Isis outcomes), the Arabic version of PDQ-39 (Tunisia) was culturally adapted and changed to an Egyptian version to avoid some words that were not familiar with the Egyptian culture. According to the standard methodology (24), two Egyptian native speakers reviewed the Tunisian Arabic version and identified terms that were unfamiliar separately. The comments of the two reviewers were revised by a third Egyptian individual to confirm their acceptability and cultural relevance. Finally, back-translation of revised items from the Egyptian Arabic version to English was performed to ensure accuracy, followed by a cognitive debriefing with 10 Egyptian patients to confirm that the new version is well understood.

### Statistical Analyses

Statistical analyses were performed using the SPSS (version 23 for windows). Categorical data, such as the frequency of NMSs, were described using frequencies and percentages, while continuous data were expressed as mean and standard deviation (SD) values. The comparison between two independent variables was made using the independent *t*-test, while the Spearman’s rank correlation coefficient was used to evaluate the association between PDQ-39 dimensions, NMSS domains, and other variables. Moreover, we used the multiple linear regression analysis to determine the predictors of PDQ-SI. The level of statistical significance (*p*-value) was set at 0.05.

## Results

### Clinical and Demographic Characteristics of PD Patients

Ninety-seven PD [58 males (59.8%) and 39 females (40.2%)] patients were enrolled in this cross-sectional study (mean age at enrollment: 55.3 ± 10.8 years and mean age of disease onset: 50.1 ± 11.2 years). The mean disease staging in the Off state (H&Y Off scale) was 2.8 ± 1.1 (stage 1: 6.4%; 1.5: 12.8%; 2: 13.8%, 2.5: 11.7; 3: 34%; 4: 12.8%; 5: 8.5%), while the mean motor severity in the Off state (UPDRS III Off) was 38.04 ± 19.37. The prevalence of depression was 76.7% in the enrolled cohort (Table 1). Females had younger age at study enrollment (*p* = 0.007) and at disease onset (*p* = 0.004), as well as worse S&E Off scores (*p* = 0.01); however, males and females had comparable disease duration, staging, and UPDRS scores.

**Table 1:** Demographic and clinical motor characteristics of PD patients.

	Mean (SD)	Range
Number	97	
Gender		
Male, *n* (%)	58 (59.8)	
Female, *n* (%)	39 (40.2)	
Age (years)	55.3 (10.8)	23–77
Duration of illness (years)	5.3 (4.1)	0.3–20
Age of onset (years)	50.1 (11.2)	20–70
H&Y off	2.78 (1.1)	1–5
H&Y On	1.00 (0.8)	0–3
S&E Off	56.0 (22)	10–90
S&E On	86.38 (13.9)	40–100
UPDRS I	3.83 (2.87)	0–12
UPDRS II off	17.88 (10.8)	0–44
UPDRS II on	7.38 (8.1)	0–41
UPDRS III off	38.04 (19.4)	4–86
UPDRS III on	15.93 (14.0)	0–92
BDI	17.5 (8.7)	

*Data presented are the mean ± SD and range unless otherwise indicated*.

*BDI, Beck depression inventory; H&Y, Hoehn and Yahr scale; S&E, Schwab and England scale; UPDRS, unified Parkinson’s disease rating scale; PD, Parkinson’s disease*.

### NMSs Prevalence and Correlations

**Figure 1:**
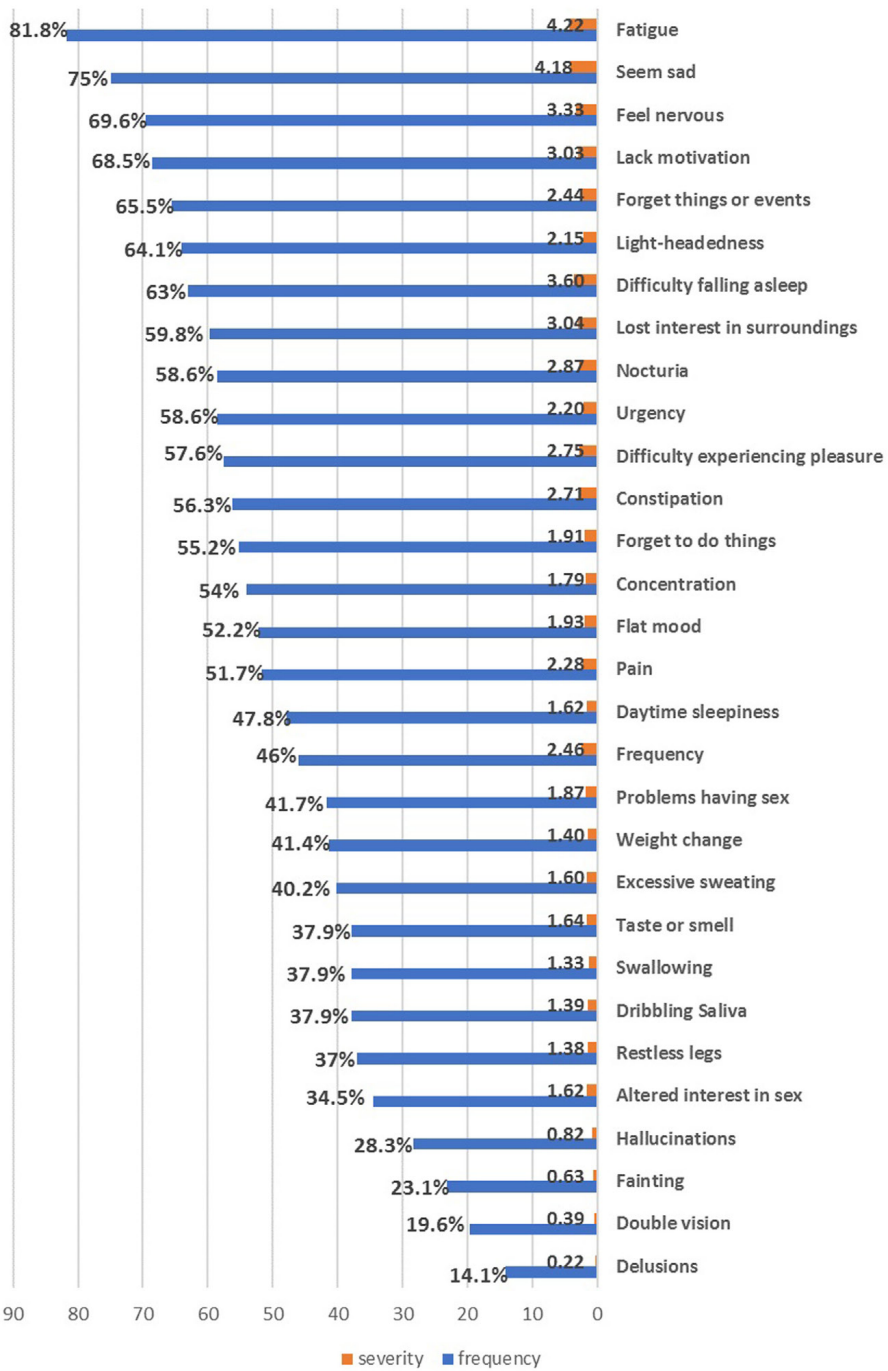
The frequency and severity of individual non-motor symptoms in the enrolled Parkinson’s disease patients.

All patients (100%) suffered from one or more NMSs. The most common and severely affected domains were sleep/fatigue (91.3%), mood/cognition (87%), miscellaneous symptoms (78.2%), urinary symptoms (75.9%), and memory/attention impairment (72.4%) (Table 2). Fatigue was the most frequent NMS (81.8%), followed by mood symptoms (sadness 75%, nervousness 69.6%, and lack of motivation 68.5%), forgetfulness (65.5%), and urinary symptoms (nocturia and urgency 58.6%) (Figure 1). No gender differences were detected except that females had worse miscellaneous domain scores (*p* = 0.018, females experienced more pain and sweating) and higher BDI scores (*p* = 0.012).

**Table 2:** Non-motor and HRQoL characteristics of enrolled PD patients.

	Mean (SD)	Frequency (%)
**Non-motor**		
NMSS total	61 (42.9)	100
Cardiovascular	2.7 (3.9)	64.8
Sleep/fatigue	10.98 (8.7)	91.3
Mood	18.2 (16.2)	87
Perceptual problems	1.4 (3)	37
Attention/memory	6.1 (6.8)	72.4
Gastrointestinal	5.4 (6.98)	67.8
Urinary	7.3 (9.5)	75.9
Sexual function	3.4 (5.96)	43.5
Miscellaneous	6.7 (6.7)	78.2
**HRQoL**		
PDQ-39 SI	37.5 (18.6)	100
Mobility	46.8 (29.92)	98.9
ADL	44.4 (28.1)	97.8
Emotional well-being	45.1 (30.1)	95.5
Stigma	50 (31.7)	86.4
Social support	18.3 (24.6)	56.8
Cognition	27.05 (20.94)	88.6
Communication	26.6 (25.2)	73.9
Bodily discomfort	40.15 (24.9)	94.3

*Data presented are mean ± SD*.

*HRQoL, health-related quality of life; NMSS, non-motor symptoms scale; PDQ-39 SI, Parkinson’s disease questionnaire-summary index; ADL, activities of daily living; PD, Parkinson’s disease*.

The total NMSS score was significantly correlated with S&E Off, UPDRS I, UPDRS II (Off & On), UPDRS III (Off & On), and BDI scores, but not with age, duration of the disease, or H&Y staging (*r* = 0.204, *p* = 0.06). Most of NMSs domains (except sexual and urinary domains) were correlated with UPDRS-I and II Off scores (Table A in Supplementary Material).

### Quality of Life State and Correlations

All enrolled PD patients had impaired HRQoL with comparable values (scores between 40 and 50) and frequency (88.6–98.9%) in most dimensions, except for social support, cognition, and communication (had lower scores, indicating better HRQoL aspects) (Table 2). About 86% of PD patients felt stigmatized to some degree, while 56.8% of the cohort reported incomplete social support. Females showed significantly more impaired (higher values) PDQ-SI, mobility, ADL, and bodily discomfort scores (*p* = 0.016, 0.001, 0.003, and 0.047, respectively).

The PDQ-SI score was inversely correlated with the patients’ age at enrollment (*r* = −0.220, *p* = 0.04), age of disease onset (*r* = −0.272, *p* = 0.01) and S&E daily activity scores (Off & On scores) (*r* = −0.483 and −0.361, *p* < 0.01, and 0.01, respectively) (Table B in Supplementary Material). This means that younger age at enrollment or disease onset and less independence in daily activity were associated with more impaired HRQoL. In addition, the PDQ-SI was positively correlated with female gender, H&Y (Off and On) disease stage (*r* = 0.318 and 0.298, *p* = 0.003 and 0.006, respectively), UPDRS-I and UPDRS-II Off (*p* < 0.001), UPDRS-III (Off and On) scores (Table 3). Thus, HRQoL impairment was associated with more impaired cognition, daily activity, and “Off and On” motor features. The disease duration did not correlate with PDQ-SI; however, it was correlated with ADL and Stigma dimensions (*r* = 0.226, 0.229; *p* = 0.030, 0.036, respectively).

**Table 3:** Correlations between quality of life domains and disease stage, patient disability and UPDRS subscores.

		PDQ-39 SI	Mobility	ADL	Emotional well-being	Stigma	Social support	Cognition	Bodily discomfort	Communication
H&Y off	*r*	0.318	0.281	0.438	0.153	0.168	0.051	0.151	0.118	0.449
	*p*	0.003	0.008	<0.001	0.160	0.121	0.638	0.165	0.279	<0.001
H&Y On	*r*	0.298	0.291	0.340	0.230	0.096	−0.138	0.249	0.098	0.380
	*p*	0.006	0.006	0.001	0.034	0.383	0.209	0.022	0.372	<0.001
S&E On	*r*	−0.361	−0.414	−0.402	−0.269	−0.167	−0.001	−0.208	−0.130	−0.305
	*p*	0.001	<0.001	<0.001	0.013	0.127	0.992	0.057	0.237	0.005
S&E Off	*r*	−0.438	−0.433	−0.463	−0.294	−0.285	−0.099	−0.252	−0.214	−0.375
	*p*	<0.001	<0.001	<0.001	0.006	0.007	0.364	0.018	0.047	<0.001
UPDRS I	*r*	0.599	0.514	0.495	0.612	0.338	0.234	0.459	0.377	0.452
	*p*	<0.001	<0.001	<0.001	<0.001	0.002	0.032	<0.001	<0.001	<0.001
UPDRS II off	*r*	0.548	0.539	0.552	0.467	0.335	0.122	0.270	0.410	0.390
	*p*	<0.001	<0.001	<0.001	<0.001	0.003	0.282	0.016	<0.001	<0.001
UPDRS II on	*r*	0.217	0.209	0.178	0.189	0.085	0.048	0.180	0.108	0.302
	*p*	0.057	0.061	0.112	0.098	0.457	0.675	0.115	0.348	0.007
UPDRS III off	*r*	0.384	0.415	0.426	0.280	0.208	0.004	0.169	0.217	0.375
	*p*	<0.001	<0.001	<0.001	0.011	0.061	0.971	0.129	0.051	0.001
UPDRS III on	*r*	0.283	0.276	0.275	0.202	0.076	0.042	0.276	0.079	0.364
	*p*	0.010	0.010	0.010	0.067	0.492	0.703	0.012	0.478	0.001

*Data presented are *p* values and correlation coefficients (*r*)*.

*H&Y, Hoehn and Yahr scale; S&E, Schwab and England scale; UPDRS, unified Parkinson’s disease rating scale; ADL, activities of daily living*.

Moreover, PDQ-SI was strongly correlated to the total and most of NMS subscales’ scores (except CVS and sexual domains) and BDI score (*p* < 0.001). The total NMSS score was strongly correlated to PDQ-39 dimensions (*p* < 0.001), except social support. Likewise, most of the PDQ-39 dimensions had a significant correlation with most of NMSS domains, except social support (Table 4). In summary, the more severe and frequent NMSs are, the more impaired HRQoL becomes in PD patients.

**Table 4:** Correlations between NMSs and quality of life domains.

		Mobility	ADL	Emotional well-being	Stigma	Social support	Cognition	Communication	Bodily discomfort	PDQ-39 SI
CVS	*r*	0.221	0.150	0.237	0.079	0.027	0.242	0.197	0.154	0.198
	*p*	0.039	0.163	0.030	0.475	0.807	0.026	0.072	0.161	0.071
Sleep and fatigue	*r*	0.643	0.548	0.563	0.389	0.148	0.508	0.559	0.415	0.659
	*p*	<0.001	<0.001	<0.001	<0.001	0.176	<0.001	<0.001	<0.001	<0.001
Mood and cognition	*r*	0.518	0.444	0.742	0.423	0.182	0.501	0.471	0.531	0.681
	*p*	<0.001	<0.001	<0.001	<0.001	0.096	<0.001	<0.001	<0.001	<0.001
Perception	*r*	0.202	0.164	0.348	0.013	0.007	0.382	0.234	0.412	0.263
	*p*	0.058	0.125	0.001	0.905	0.953	<0.001	0.031	<0.001	0.015
Memory	*r*	0.289	0.303	0.378	0.137	0.242	0.669	0.404	0.174	0.421
	*p*	0.008	0.005	0.001	0.223	0.029	<0.001	<0.001	0.120	<0.001
GIT	*r*	0.455	0.343	0.433	0.148	0.049	0.474	0.415	0.466	0.452
	*p*	<0.001	0.001	<0.001	0.188	0.666	<0.001	<0.001	<0.001	<0.001
Urinary	*r*	0.333	0.359	0.383	0.182	0.099	0.308	0.335	0.340	0.414
	*p*	0.002	0.001	<0.001	0.104	0.380	0.005	0.002	0.002	<0.001
Sexual	*r*	0.060	0.164	0.217	0.035	0.154	0.261	0.201	0.053	0.151
	*p*	0.593	0.140	0.054	0.760	0.176	0.020	0.076	0.643	0.184
Total	*r*	0.625	0.573	0.697	0.400	0.160	0.613	0.594	0.526	0.723
	*p*	<0.001	<0.001	<0.001	<0.001	0.155	<0.001	<0.001	<0.001	<0.001
BDI	*r*	0.671	0.578	0.745	0.524	0.309	0.569	0.574	0.387	0.765
	*p*	<0.001	<0.001	<0.001	<0.001	0.005	<0.001	<0.001	<0.001	<0.001

*Data presented are *p* values and correlation coefficients (*r*)*.

*ADL, activities of daily living; BDI, Beck depression inventory; CVS, cardiovascular; GIT, gastrointestinal; PDQ-SI, Parkinson’s disease questionnaire-summary index; NMSs, non-motor symptoms*.

Multiple linear regression analysis was performed to determine the predictors of the PDQ-SI from different variables including age at enrollment and disease onset, gender, disease duration, UPDDRS I, II, III, BDI, H&Y, S&E, and total NMSS scores. Depression (BDI) and the total NMSS score explained 50.5% of the PDQ-SI. Depression was the primary predictor (adjusted *R*^2^ 0.497, *p* < 0.0001). On removing BDI score from the model, the total NMSS score and UPDRS-I (cognition) were the independent predictors of HRQoL and explained 53.7% of patients’ HRQoL. The total NMSS score was the primary predictor (adjusted *R*^2^: 0.480, *p* < 0.0001).

## Discussion

This study explored the non-motor and HRQoL profiles of PD patients in an African country with a high prevalence of PD. It confirmed the marked impact of NMSs and depression on HRQoL impairment. Moreover, it provides a culturally adapted Arabic version of the PDQ-39 to promote further research and care of Arabic PD patients. To the best of our knowledge, this is the first study to investigate the HRQoL and its association with NMSs in Egyptian PD patients.

Our study showed a high prevalence of NMSs in Egyptian PD patients (100%). Fatigue, mood disturbances (sadness, nervousness, and lack of motivation), and memory impairment were the most frequent NMSs. Likewise, the most severe and frequently affected domains were sleep/fatigue and mood/cognition domains. Gender differences were minimal and restricted to miscellaneous symptoms. A similar high prevalence of NMSs and frequency of NMS domains were reported in Upper Egypt by Khedr and colleagues (16). This NMS profile in the Egyptian patients is more consistent with the depression/anxiety PD subtype (11).

Previous studies have identified differences in NMS profile between different countries and races. Reports from different Asian countries identified GIT symptoms, especially constipation as the most frequent NMS (8). Nocturia was the most frequent NMS, followed by fatigue and dribbling of saliva in a multicenter European study (3). Cognition, sleep followed by urinary symptoms were the most common NMS in an Estonian cohort (25), while fatigue followed by urinary symptoms were more common in the Spanish population (26). These differences could be attributed to several demographic, genetic, and clinical variations in enrolled patients (age, the age of onset, disease duration, and severity), as well as different assessment questionnaires (10, 12).

The different dimensions of HRQoL were impaired in all patients and markedly correlated to NMSS total score and depression severity. Moreover, it was associated with motor severity, disease staging, and impaired daily activity in Off and On states. However, regression analysis confirmed the independent predicting effect of NMSs for HRQoL. This is consistent with the findings of former studies in different populations in which the total NMSS score was the main predictor of QoL. Mood/cognition and sleep/fatigue had the strongest correlation with PDQ-SI in accordance with prior studies (3, 7, 27).

Neuropsychiatric symptoms, especially depression, were reported as independent determinants of HRQoL (28). In consistence with previous studies (25, 26, 29), depression was recognized as the main independent predictor of HRQoL. This could be explained by the high prevalence of depression (76.7%) and the strong correlation between depression and all HRQoL dimensions, several NMS domains and total NMSS scores. Consequently, depression affects HRQoL directly and indirectly through its impact on the NMSs, especially emotional well-being and cognition (28). In agreement with several previous studies, depression was recognized as the main predictor of poor HRQoL in PD patients (29, 30). Antonini et al. reported improvement in mobility and ADL after depression treatment, indicating that mood problems affect how PD patients perceive their motor functions (31).

Intriguingly, 86.4% of PD patients felt stigmatized, while 56.8% of the cohort reported incomplete social support. Stigma was associated with younger age of enrollment, younger age of disease onset, disease severity, impaired daily activities and cognition, as well as depression. This is consistent with prior studies (32, 33). Furthermore, it was correlated with fatigue/memory, mood/cognition domains, and total NMSS scores. Stigma is an important determinant of HRQoL, related to cultural and social factors (32, 34). Consequently, the identification and management of this feature are crucial for the improvement of HRQoL of PD patients.

Although this study provides a comprehensive assessment of the impact of different NMS domains on HRQoL for the first time in Egyptian patients, it has some limitations. The relatively small sample size and the fact that the majority of enrolled patients came from a low socioeconomic class restrict the generalizability of our findings. Moreover, as a clinic-based study [plus the relatively short disease duration (5.3 years)], patients with advanced stages and cognitive impairment were under-represented. Furthermore, some NMSs are prevalent in the elderly population without PD; therefore, a case–control study would elucidate whether the investigated NMSs are PD related.

In conclusion, this study demonstrated the high prevalence of NMSs in Egyptian PD patients and the significant impact of NMSs and depression on HRQoL. Thus, the assessment and management of NMSs are as important as the motor aspects of PD. Future larger studies can use our culturally adapted questionnaire to evaluate the correlation between NMSs domains and HRQoL dimensions in patients with advanced PD.

## Ethics Statement

The ethical committee of the faculty of medicine, Ain Shams University has approved this study. The authors assert that all procedures contributing to this work comply with the ethical standards of the relevant national and institutional committees on human experimentation and with the Helsinki Declaration of 1975, as revised in 2008. All subjects gave written informed consent according to the Declaration of Helsinki.

## Author Contributions

All authors made substantial contributions, revised the manuscript, and approved the final version. AS, HE, SA, and ME: conception and design of the study. AS, AB, MH, MK, and EH: data collection and analysis. AS, AB, AA, and HE: first draft writing. All authors: revision of the manuscript.

## Conflict of Interest Statement

The authors declare that the research was conducted in the absence of any commercial or financial relationships that could be construed as a potential conflict of interest.

## Abbreviations

ADL, activities of daily living; BDI, Beck depression inventory; HRQoL, health-related quality of life; H&Y, Hoehn and Yahr scale; NMSs, non-motor symptoms; NMSS, non-motor symptoms scale; PD, Parkinson’s disease; PDQ-SI, Parkinson’s disease questionnaire-summary index; S&E, Schwab and England scale; UPDRS, unified Parkinson’s disease rating scale.
