# Supplementary material for: Non-Motor Symptoms as Predictors of Quality of Life in Egyptian Patients With Parkinson’s Disease: A Cross-Sectional Study Using a Culturally Adapted 39-Item Parkinson’s Disease Questionnaire
Source: Front Neurol. 2018 May 24;9:357. doi: 10.3389/fneur.2018.00357 (PMC5976737; doi:10.3389/fneur.2018.00357)
Supplement: Supplementary file 1 [file Table_1.docx]

**Supplementary table A: Correlation between demographic data, motor and non-motor symptoms**

|  |  | **CVS** | **Sleep** | **Mood** | **Perception** | **Memory** | **GIT** | **Urinary** | **Sexual** | **Total** |
| --- | --- | --- | --- | --- | --- | --- | --- | --- | --- | --- |
| **Age** | *r* | 0.01 | -0.07 | -0.16 | 0.08 | 0.144 | 0.22 | 0.204 | 0.111 | 0.01 |
|  | *p* | 0.9 | 0.53 | 0.134 | 0.451 | 0.185 | 0.04 | 0.058 | 0.313 | 0.93 |
| **DOI (years)** | *r* | -0.13 | 0.18 | 0.084 | -0.1 | -0.017 | 0.19 | 0.069 | 0.157 | 0.12 |
|  | *p* | 0.22 | 0.09 | 0.428 | 0.341 | 0.873 | 0.07 | 0.525 | 0.152 | 0.29 |
| **AOO** | *r* | 0.05 | -0.11 | -0.18 | 0.093 | 0.114 | 0.14 | 0.167 | 0.058 | -0.03 |
|  | *p* | 0.599 | 0.259 | 0.072 | 0.376 | 0.294 | 0.185 | 0.123 | 0.596 | 0.748 |
| **H&Y off** | *r* | 0.064 | 0.273 | 0.185 | 0.036 | 0.097 | 0.192 | 0.035 | 0.112 | 0.204 |
|  | *p* | 0.553 | 0.01 | 0.082 | 0.738 | 0.378 | 0.08 | 0.753 | 0.315 | 0.062 |
| **H&Y On** | *r* | 0.078 | 0.209 | 0.172 | 0.21 | 0.126 | 0.212 | 0.018 | -0.174 | 0.204 |
|  | *p* | 0.471 | 0.049 | 0.106 | 0.049 | 0.254 | 0.052 | 0.874 | 0.116 | 0.063 |
| **S&E On** | *r* | -0.084 | -0.166 | -0.184 | -0.062 | -0.101 | -0.156 | -0.187 | 0.06 | -0.203 |
|  | *p* | 0.438 | 0.121 | 0.084 | 0.562 | 0.361 | 0.157 | 0.088 | 0.588 | 0.065 |
| **S&E Off** | *r* | -0.061 | -0.461 | -0.33 | 0.019 | -0.161 | -0.132 | -0.101 | -0.02 | -0.347 |
|  | *p* | 0.568 | <0.001 | 0.002 | 0.863 | 0.142 | 0.229 | 0.36 | 0.856 | 0.001 |
| **UPDRS I off** | *r* | 0.232 | 0.572 | 0.568 | 0.456 | 0.302 | 0.318 | 0.211 | 0.128 | 0.562 |
|  | *p* | 0.029 | <0.001 | <0.001 | <0.001 | 0.005 | 0.003 | 0.052 | 0.249 | <0.001 |
| **UPDRS II off** | *r* | 0.25 | 0.549 | 0.528 | 0.286 | 0.217 | 0.306 | 0.207 | 0.083 | 0.524 |
|  | *p* | 0.026 | <0.001 | <0.001 | 0.01 | 0.057 | 0.007 | 0.071 | 0.48 | <0.001 |
| **UPDRS II on** | *r* | 0.188 | 0.253 | 0.168 | 0.203 | 0.22 | 0.183 | 0.124 | 0.077 | 0.262 |
|  | *p* | 0.091 | 0.022 | 0.131 | 0.068 | 0.051 | 0.106 | 0.278 | 0.504 | 0.02 |
| **UPDRS III off** | *r* | 0.109 | 0.389 | 0.283 | 0.171 | 0.094 | 0.057 | 0.051 | -0.032 | 0.247 |
|  | *p* | 0.326 | <0.001 | 0.01 | 0.122 | 0.413 | 0.618 | 0.656 | 0.783 | 0.029 |
| **UPDRS III on** | *r* | 0.219 | 0.268 | 0.159 | 0.23 | 0.264 | 0.166 | 0.127 | 0.12 | 0.294 |
|  | *p* | 0.043 | 0.012 | 0.14 | 0.032 | 0.016 | 0.135 | 0.254 | 0.287 | 0.007 |
| **BDI** | *r* | 0.155 | 0.691 | 0.687 | 0.163 | 0.401 | 0.341 | 0.198 | 0.248 | 0.715 |
|  | *p* | 0.164 | <0.001 | <0.001 | 0.141 | <0.001 | 0.002 | 0.078 | 0.028 | <0.001 |

**Supplementary table B: Correlation between demographic criteria and quality of life domains**

|  |  | **PDQ39 SI** | **Mobility** | **ADL** | **Emotional wellbeing** | **Stigma** | **Social support** | **Cognition** | **Bodily discomfort** | **Communication** |
| --- | --- | --- | --- | --- | --- | --- | --- | --- | --- | --- |
| **Age** | ***r*** | -0.22 | -0.113 | -0.02 | -0.204 | -0.43 | -0.14 | 0.015 | -0.075 | -0.122 |
|  | ***p*** | 0.04 | .0285 | 0.83 | 0.057 | <0.00 | 0.20 | 0.886 | 0.486 | 0.257 |
| **Gender** | ***r*** | 0.24 | 0.439 | 0.31 | 0.176 | 0.14 | -0.07 | 0.094 | 0.210 | -0.079 |
|  | ***p*** | 0.02 | <0.00 | 0.00 | 0.101 | 0.17 | 0.47 | 0.384 | 0.050 | 0.464 |
| **DOI (years)** | ***r*** | 0.17 | 0.133 | 0.22 | 0.008 | 0.23 | 0.07 | -0.004 | 0.061 | 0.198 |
|  | ***p*** | 0.11 | 0.205 | 0.03 | 0.942 | 0.03 | 0.53 | 0.968 | 0.571 | 0.064 |
| **AOO** | ***r*** | -0.27 | -0.163 | -0.10 | -0.220 | -0.47 | -0.19 | -0.005 | -0.077 | -0.193 |
|  | ***p*** | 0.01 | 0.122 | 0.34 | 0.039 | <0.001 | 0.07 | 0.963 | 0.474 | 0.071 |

**Abbreviations:** AOO: age of onset, ADL: Activities of daily living, BDI: Beck Depression Inventory, CVS: Cardiovascular system, DOI: Duration of illness, GIT: Gastrointestinal, HRQoL: Health related-quality of life, H&Y: Hoehn & Yahr scale, NMS: Non-motor symptoms, NMSS: Non-motor symptoms scale, PDQ-SI: Parkinson's' disease questionnaire-summary index, S&E: Schwab & England UPDRS: Unified Parkinson's disease rating scale.
